# Determination of the association between the levels of physical activity and cognitive perceptions of physicians and the frequency of family doctors prescribing exercise

**DOI:** 10.1017/S1463423626101121

**Published:** 2026-04-20

**Authors:** Kübra Kurt, Pınar Döner Güner

**Affiliations:** 1 Hatay Arsuz State Hospital, Turkey; 2 Department of Family Medicine, Hatay Mustafa Kemal University Tayfur Ata Sökmen Faculty of Medicine, Turkey

**Keywords:** Cognitive behavior, exercise prescription, primary care

## Abstract

**Aim:**

The aim of this study was to determine the relationship between the physical activity levels and cognitive perceptions of physicians and the frequency of exercise prescribed by primary care physicians.

**Methods:**

This cross-sectional study was conducted with 221 primary care physicians. A questionnaire of three sections was administered, including questions prepared according to the American College of Sports Medicine (ACSM) recommendations evaluating the current practices of the physicians on the subject of prescribing exercise, the General Practice Physical Activity Questionnaire (GPPAQ), Cognitive Behavioural Physical Activity Questionnaire (CBPAQ).

**Results:**

A significant relationship was determined between the daily physical activity of the physician and exercise prescribing rates (*P* = 0.005). From the data obtained from the GPPAQ and the CBPAQ, it was determined that as the activity level increased, so the Outcome Expectation (*P* < 0.001), Self-regulation (*P* < 0.001), Total Cognitive Activity (*P* < 0.001) points increased. The frequency of prescribing exercise was found to be <20% for all chronic diseases for which exercise is known to be effective. A significant relationship was determined between prescribing exercise and the total number of correct responses to the questions measuring the level of knowledge according to the ACSM recommendations (*P* < 0.001). Mann-Whitney U and Kruskal-Wallis tests were used for non-normally distributed data, while Pearson, likelihood ratio, and chi-square tests were used for analyzing relationships between categorical variables.

**Conclusion:**

Incorporating exercise prescription training into the core medical and family medicine curricula may increase physicians’ self-efficacy and contribute to overcoming barriers in prescribing exercise.

## Background

An exercise prescription refers to much more than an implicit recommendation for exercise, as it requires sufficient specificity, detail and depth, and therefore very good knowledge of the patient profile (Chen et al., 2018; Luan et al., 2019). There are many underlying reasons for exercise not going beyond a recommendation. Many studies have been conducted throughout the world to determine these reasons, and according to the results of those studies, the most common reasons are that the physician cannot allocate enough time for the patient and that the physician does not feel sufficiently competent to prescribe exercise, has insufficient resources, and does not show sufficient interest in the patient (Douglas et al., 2006; Parker et al., 2011; Hébert et al., 2012; Florindo et al., 2013).

Although exercise is free of charge and as effective as drugs, for which hundreds of millions of dollars are paid each year, it is still not sufficiently prescribed for the treatment of chronic diseases, and it is not fully followed by patients as prescribed. It has also been seen that the promotion of exercise to patients within lifestyle changes has lagged behind other recommendations such as smoking, diet, and alcohol (Bock et al., 2012; Lindsley 2014; López-Román et al., 2020). Recommendations have been made by the World Health Organization (WHO) and ACSM giving information about the basic components of exercise with the abbreviation FITT (frequency, intensity, time, type) for healthy individuals and patients. This was done with the aim of resolving the problem of a sedentary lifestyle, which is a modifiable risk factor, and to increase the prescription of exercise as a medication, which would contribute to the economy of the country (Alwan, 2011).

In a study in Canada, it was reported that 68% of doctors gave verbal counseling and recommendations for exercise, and only 15.8% used written exercise prescriptions (Petrella et al., 2007). In Türkiye there are no guidelines, but there are a few studies showing the difference between the existing and desired situation on the subject of primary care physicians counseling patients and actually writing exercise prescriptions. (Petrella et al., 2007; Lobelo et al., 2009; Windt et al., 2015). There remains a need for current research to be developed in respect of determining the reasons that exercise prescribing is not at the desired level.

When the population served by a physician is considered in terms of patient profile, primary care physicians are in a unique position to be able to write personalized exercise prescriptions with the advantages of being a continuous point of contact and having all the detailed information about the patient (Solmundson et al., 2016; Luan et al., 2019). Therefore, the aim of this study was to determine the frequency of exercise prescribing by primary care physicians, their current practices, and the barriers to prescribing exercise. It was also thought that the cognitive perceptions of physicians could affect motivational approaches and their own lifestyle habits could have an effect on their exercise-prescribing practices.

In this context, it was aimed to first measure the frequency of exercise prescribing of physicians, their cognitive perceptions on the subject of exercise, and their own levels of physical activity. The secondary aim was to determine the relationships between the frequency of exercise prescribing and the cognitive perceptions and daily physical activity levels of the physicians.

## Methods

### Permission for the research, location, and dates

Approval for this descriptive, cross-sectional study was granted by the Non-Interventional Research Ethics Committee of Hatay Mustafa Kemal University (decision no. 14, session no. 12, dated, 01 November 2021). The necessary permission to conduct the study was obtained from Hatay Province Health Directorate.

The study was conducted between 1 December, 2021, and 30 April, 2022, in all the primary care centers in the cities, towns, and villages of Hatay province.

### Research universe and sample size

In a pilot study conducted on 50 subjects to test the comprehensibility of the questionnaire questions and calculate the sample size, the frequency of exercise prescribing was seen to be 42%. According to this rate, from the central population of 505 primary care physicians, it was calculated to be necessary to have a minimum sample of 216 subjects to provide a 95% confidence interval and 5% error margin. Taking potential data losses into consideration, it was decided that the minimum number should be 220. All 505 primary care family doctors in the province of Hatay were contacted, and a total of 240 voluntarily agreed to participate in the study.

The pilot study, conducted with 50 participants to test feasibility and measurement procedures, was included in the main analysis in line with standard methodological practice, as no modifications were made to the study protocol.

The questionnaires of 19 respondents were not completed in full, so were excluded from the analyses. Consequently, the study was completed with the data of 221 primary care physicians.

### Study protocol

The study questionnaires were applied to physicians actively working in polyclinic services within working hours at times when there were no patients. All the physicians provided informed consent before completing the study questionnaires. In Hatay Centre and nearby towns, the questionnaires were administered face-to-face. For the physicians in all the villages and in 5 towns far from the center, a link to the questionnaire together with a written explanation was sent online (e-mail, WhatsApp) to the primary care units.

After 2 reminders via e-mail and WhatsApp sent at 2-week intervals, fully completed questionnaires were received from 221 primary care physicians: 133 completed face-to-face and 88 online.

The questionnaire comprised 3 sections: CBPAQ, GPPAQ, and questions prepared according to information in the relevant literature.

The GPPAQ consists of 7 questions under 3 main headings of activity in the workplace, activities performed in the last 7 days and how many hours per week, and the speed of normal walking. The scale measures the overall level of activity in 4 categories as active, moderately active, slightly active, and inactive.

The CBPAQ was designed to determine attitudes and behavior towards participation in exercise. The questionnaire comprises 15 items scored on a 5-point Likert-type scale, in 3 subsections of Outcome Expectation (OE), Self-Regulation (SR), and Personal Barriers (PB). OE shows the benefit expected as a result of participation. For example, ‘I will feel physically better after performing exercise’. SR shows the self-motivational approaches to how exercise can be included in their lifestyle. For example, ‘I schedule the activities in my life according to my exercise habits’. PB indicates the barriers to performing exercise placed by the subject. For example, ‘I cannot find the time for exercise as I have too much work to do all day’. The total score is the total of points obtained from each section and higher values indicate higher cognitive perceptions in the relevant area.

The questionnaire prepared as a result of the literature scan comprised 33 items: 17 questions to elicit sociodemographic information (age, gender, years of professional experience) and knowledge on the subject of prescribing exercise and 16 questions related to the knowledge content prepared according to the ACSM recommendations.

### Statistical analysis

Data obtained in the study were analyzed statistically using SPSS version.21 software. Conformity of the numerical data to normal distribution was examined with visual and analytical methods. Descriptive analyses were reported as mean ± standard deviation (SD) values for variables with normal distribution and as median, minimum, and maximum values for data not showing normal distribution. In the inter-group comparisons, the Mann-Whitney U test and Kruskal-Wallis test were used for data not showing normal distribution. Relationships between categorical variables were analyzed using Pearson, likelihood ratio, and chi-square tests. The Cronbach alpha value for the questions measuring the knowledge level according to the ACSM recommendations was calculated as 0.89. A value of *P* < 0.05 was accepted as the level of statistical significance in all the tests.

## Results

The data were evaluated from a total of 221 primary care physicians, comprising 152 (68.8%) males and 69 (31.2%) females with a mean age of 44.8 ± 8.9 years (males: 45.81 ± 9.06, females: 42.65 ± 8.13) and mean duration of professional experience of 19.24 ± 8.98 years (range, 2–38 years).

It was determined that 29.4% of the physicians often provided exercise counseling and 10% often prescribed exercise.

According to the results of the GPPAQ, in the evaluation of the physical activity level groups, the largest group was the inactive group at the rate of 50.2% (*n*: 111) (Table 1). When the relationship between physicians’ daily physical activity levels and exercise prescription rates was examined, physicians in the slightly active group were more likely not to prescribe exercise than to prescribe it (58.8% vs. 35.9%). In contrast, physicians in the moderately active and active groups prescribed exercise more frequently than they did not prescribe; in the moderately active group, 47.1% prescribed exercise compared with 37.3% who did not, while in the active group the corresponding rates were 17.1% and 3.9%, respectively. These differences were statistically significant (chi-square test, *P* = 0.005).

**Table 1.** Physicians’ physical activity levels according to the BBFAA and their perceived competence in exercise prescription

		***n***	**%**
**Physicians’ perceived self-efficacy in exercise prescription.**	**Yes**	19	8.6
	**No**	106	**48.0**
	**Partially**	96	43.4
**GPPAQ categories**	**Inactive**	111	**50.2**
	**Slightly active**	56	25.3
	**Moderately active**	35	15.8
	**Active**	19	8.6

n: none.

According to the CBPAQ data, the highest points were obtained in the OE section (3.63 ± 1.43) and the lowest points in the SR section (2.62 ± 1.17). The mean CBPAQ total points of the physicians were determined to be 3.53 ± 2.16. When the relationship was examined between the exercise-prescribing behavior of the physicians and the OE, SR, and PB subsections of cognitive perceptions, it was determined that the physicians with higher SR and total cognitive perception points and lower PB points prescribed exercise more (Table 2).

**Table 2.** The relationships between exercise prescribing and the CBPAQ points

		**Exercise prescribing**		***P***
**CBPAQ**		**Yes**	**No**	
**Outcome expectation**	**Median**	4	4.2	0.398
	**Min-Max**	1.00–5.00	1.00–5.00	
**Self-regulation**	**Median**	2.8000	2.2000	0.01
	**Min-Max**	1.00–5.00	1.00–5.00	
**Personal barriers**	**Median**	2.80000	3.40000	0.02
	**Min-Max**	1.00–5.00	1.00–4.6	
**Total**	**Median**	3.6000	3.0000	0.049
	**Min-Max**	1.00–9.00	0.2–8.00	

Min: minimum.

Max: maximum.

The relationship between the CBPAQ scores and the GPPAQ scores was examined, and it was determined that as the activity level increased, so the OE, SR, and total cognitive behavior points increased (*P* < 0.001). Thus it was seen that when the benefits to be obtained from exercise, motivational regulation for a life in which exercise can be included, and the total cognitive perception on this subject increased, so the activity level of the physicians also increased. Correspondingly, as the activity level of the physicians increased, there was found to be a decrease in the PB scores (*P* = 0.135) (Table 3).

**Table 3.** The relationships between the CBPAQ and the GPPAQ

	**GPPAQ**								***P***
	**Inactive [** ***n*** **= 111]**		**Slightly active [** ***n*** **= 56]**		**Moderately active [** ***n*** **= 35]**		**Active [** ***n*** **= 19]**		
	**Median**	**Min-Max**	**Median**	**Min-Max**	**Median**	**Min-Max**	**Median**	**Min-Max**	
**Outcome expectation**	4.00	1.00–5.00	4.00	1.00–5.00	4.40	1.00–5.00	4.80	1.00–5.00	<0.001
**Self-regulation**	2.20	1.00–5.00	2.50	1.00–5.00	3.40	1.00–5.00	4.00	1.00–5.00	<0.001
**Personal barriers**	3.20	1.00–5.00	2.80	1.00–5.00	2.80	1.00–5.00	1.80	1.00–5.00	0.135
**Total**	2.60	1.00–5.00	3.30	1.00–5.00	4.40	1.00–5.00	6.20	1.00–5.00	<0.001

n: none.

Min: minimum.

Max: maximum.

Only 19 (8.6%) of the physicians in this study felt competent on the subject of prescribing exercise. In Table 4, the relationships are shown between exercise prescribing and the number of correct responses given to the questions formed according to the ACSM recommendations to measure the level of knowledge about exercise prescribing (Cronbach alpha value: 0.86). The physicians who gave more correct responses were determined to write more exercise prescriptions and were also seen to feel more competent on this subject (*P* < 0.001).

**Table 4.** The relationship between the total number of correct answers and physicians’ self-efficacy in prescribing exercise

		**Total number of correct responses to the questions formed according to the ACSM recommendations, measuring the level of knowledge**		***P***
**The physician feels competent on the subject of exercise prescription**	**Yes**	**Median**	11	<0.001
		**Min-Max**	2.00–13.00	
	**No**	**Median**	6	
		**Min-Max**	0.00–14.00	
	**Partially**	**Median**	8	
		**Min-Max**	0.00–14.00	
**Exercise prescribing**	**Yes**	**Median**	8	<0.001
		**Min-Max**	0.00–14.00	
	**No**	**Median**	4	
		**Min-Max**	0.00–13.00	

Min: minimum.

Max: maximum.

The rates of providing information about the components of exercise were examined. It was seen that 68.3% provided information about the frequency of exercise, and 64.7% of the physicians did not give any information about exercise progression. When the frequency of counseling was examined, 5% of the physicians only counseled patients on first presentation (Table 5).

**Table 5.** The frequencies of physicians giving information to patients about the components of exercise and counseling

**Options**	**Categories**	***n***	**%**
**I give information about the duration of exercise**	**Yes**	141	63.8
	**No**	80	36.2
**I give information about the type of exercise**	**Yes**	146	66.1
	**No**	75	33.9
**I give information about the frequency of exercise**	**Yes**	151	68.3
	**No**	70	31.7
**I give information about the intensity of exercise**	**Yes**	121	54.8
	**No**	100	45.2
**I give information about the progression of exercise**	**Yes**	78	35.3
	**No**	143	64.7
**Frequency of counseling**	**At every presentation**	25	11.3
	**Only on first presentation**	11	5
	**Sometimes**	159	71.9
	**Never**	26	11.8

n: none.

## Discussion

### Main findings

The results of this study demonstrated that physicians with a high level of self-regulation cognitive perception on the subject of physical activity prescribed exercise more. The physicians with high SR showed a more positive approach. For example, ‘I set my own goals to be able to be physically active’ is one of the statements used to calculate self-regulation. By motivating both the physician and the patient, this positive approach may be one of the reasons increasing the rate of prescribing exercise (Molanorouzi et al., 2015). Physicians with high PB scores were found to prescribe exercise less. The effect of SR and PB perceptions on the exercise prescribing behavior of the physicians shows that exercise prescribing practices can be increased by changing perceptions of this subject. No significant relationship was found between exercise prescribing and outcome expectations, as the other subsection of the CBPAQ. These findings demonstrate the importance of physician behavior to conditions preventing the prescribing of exercise.

The results of this study showed that the frequency of counseling was extremely low, and in respect of the components of exercise, the least information was given on the subject of exercise progression. The low rates of frequency of counseling and providing information about progression by physicians who already had a low rate of prescribing exercise could be a reason for patients not continuing even if they start to exercise.

Although data from the pilot study were included in the main analysis, the decrease in exercise prescription rates from 42% in the pilot study to 10% in the main study may be explained by factors such as the larger sample size, differences in participant characteristics, and more systematic data collection in the main study; to address this potential influence, sensitivity analyses excluding the pilot data were conducted and yielded consistent results.

### Study strengths and limitations

This study can be considered of value as the first study in literature to have evaluated the effect on exercise prescribing of the physical activity levels and cognitive perceptions of physical activity of physicians. The physicians included in the study were primary care family doctors who are the first point of contact and in continuous contact with patients. Unlike other studies, the scales used were the GPPAQ and the CBPAQ. In addition, to measure the level of knowledge, the physicians were asked questions about exercise prescribing, which were prepared according to the ACSM recommendations and for which the Cronbach alpha value was calculated (Table 6). Thus, when answering the questionnaire, both the knowledge levels of the physicians and their daily practices were investigated, and the effect of the physician’s level of knowledge about exercise prescribing on daily practices was evaluated.

However, limitations of the study can be said to be primarily that the research was conducted in the single province of Hatay (south-east Türkiye), and therefore, the results cannot be generalized across the whole country. In addition, some of the questionnaires were completed online, and the reasons for not wishing to participate were unknown.

### Comparisons with current literature

Many studies have been conducted throughout the world to determine the underlying reasons that exercise does not go beyond a recommendation. The results of those studies have shown that the most common reasons are that the physician cannot allocate enough time for the patient, does not feel sufficiently competent to prescribe exercise, has insufficient resources, and does not show sufficient interest in the patient(Douglas et al., 2006; Parker et al., 2011; Hébert et al., 2012; Florindo et al., 2013). In the current study, the relationship was evaluated between the exercise-prescribing practices of the physicians and their own physical activity level and cognitive perceptions on this subject. Lawdor D.A. et al. in Canada reported that most doctors (69.8%) used verbal counseling to encourage physical activity, and only 15.8% used written prescriptions for a physical activity introductory program (Petrella et al., 2007). In the current study, the rate of verbal counseling was higher than that of written exercise prescribing, and the number of physicians prescribing exercise in written form was extremely low. These findings highlight the need for targeted training programs and supportive resources to strengthen physicians’ exercise prescription practices and increase the use of written prescriptions. Although physicians appear to be aware of the importance of exercise recommendations for patients, the persistently low rates of written exercise prescription underscore the need for qualitative research to explore the underlying barriers; such studies may help to fill this gap by identifying contextual, educational, and systemic factors that hinder effective exercise prescribing and inform strategies to overcome them.

In another questionnaire study in Germany by Christina Bock et al., related to the promotion of physical activity in primary care, it was reported that 26.9% of physicians did not have sufficient knowledge to provide counseling and 36.7% felt that they were unsuccessful in motivating patients to increase physical activity (Bock et al., 2012). F. Lobelo et al. stated that the physical activity habits, training, and motivation of doctors and medical students could be seen as barriers to exercise counseling (Lobelo et al., 2009). In a study by Johann Windt et al. that aimed to determine the number of physicians prescribing exercise after a 3-hour training session with practical prescribing tools, there was seen to be a significant increase in primary care physicians reporting that they had written physical activity prescriptions (Windt et al., 2015). In a randomized, controlled study by Sok Ying Liaw et al. and studies by Jesse Clanton et al., there was determined to be a positive correlation between self-confidence and medical knowledge of the subject (Liaw et al. 2012; Clanton et al., 2014). In support of the findings of those studies, it was seen in the current study that physicians with a high level of knowledge, measured by the questions formed according to the ACSM recommendations, felt more competent prescribing exercise, and those who felt competent prescribed exercise more.

Throughout the world in general, there are very few medical faculties in which the syllabus includes exercise medicine. In the USA, 13% of medical faculties provide education about exercise, 6% have core lessons, and 87% provide no exercise medicine training in the syllabus (Solmundson et al., 2016).

The deficiencies in the current medical faculty syllabuses in respect of exercise as medicine have been exposed. Therefore, this study can be of guidance in the provision and development of evidence-based recommendations and guidelines to facilitate exercise prescribing by primary care physicians.

F. Lobelo et al. reported that the physical activity habits of doctors and medical students affected counseling practices, and Erica Frank et al. similarly showed that the personal health practices of physicians could affect outcomes for patients (Frank 2004; Lobelo et al., 2009). In the current study, it was also determined that the physicians who stated that they were physically active prescribed exercise at a significantly higher level. This can be attributed to the fact that physically active physicians have overcome physical inactivity and are able to practically and more often implement solutions to overcome barriers to physical activity. Moreover, as they have experienced the benefits of physical activity themselves, this may be more effective, convincing, and motivating when explaining to patients.

### Implications

The relationship between exercise prescribing rates and the CBPAQ was examined in this study for the first time. The study results showed higher exercise prescribing rates of physicians with high cognitive perceptions of physical activity. This result provides a clue to how exercise prescribing rates can be increased. An increase in the cognitive perceptions of exercise could result in physicians writing more exercise prescriptions.

To increase the level of knowledge of physicians on this subject, the subject of prescribing exercise could be added to the basic medical syllabus and to the family doctor specialism education syllabus. The elimination of deficiencies in knowledge will enable physicians to feel self-confident on this subject and overcome the barrier to prescribing exercise. In addition to providing not only knowledge content but also communication skills for a motivational physician approach to determine and overcome the barriers to exercise, this education could also increase patient compliance and support the applicability of exercise prescribing. This key method could first overcome the resistance of physicians to prescribing exercise and then the resistance of patients to following the exercise prescription.

Finally, as physical inactivity is a modifiable risk factor, clinicians should routinely evaluate the structured exercise and lifestyle activities of all patients, and prescription should be made as necessary. There is a need for further studies to develop programs and guidelines for the practical implementation of exercise prescriptions and which will produce solutions to existing barriers.

## Conclusion

The results of this study demonstrated a relationship between the frequency of prescribing exercise and the physical activity level and cognitive perception of physicians. As the physical activity level and cognitive perceptions of physicians increased, so the frequency of prescribing exercise was also seen to increase. The low rates of physicians prescribing exercise was affected by the fact that physicians felt that they did not have sufficient knowledge on the subject of exercise, they had low self-confidence on this subject, and low motivation.

**Table 6.** Questions prepared in accordance with ACSM recommendations and the accuracy rates of the given answers

**Expressions used in the questions**	**Accuracy categories of the given answers**	***n***	**%**
**1: Exercises that increase cardiovascular endurance, such as walking and running, are examples of anaerobic exercises**	**True**	116	52.5
	**False**	50	22.6
	**No idea**	55	24.9
**2: Exercises aimed at increasing short-term muscle strength, such as weightlifting, are examples of aerobic exercises**	**True**	92	41.6
	**False**	70	31.7
	**No idea**	59	26.7
**3: According to the ACSM, the goal is to expend 150–400 kcal of energy through physical activity or daily exercise**	**True**	79	35.7
	**False**	13	5.9
	**No idea**	129	58.4
**4: For sedentary individuals, a low-intensity, long-duration exercise program should be initiated**	**True**	133	60.2
	**False**	32	14.5
	**No idea**	56	25.3
**5: For sedentary individuals, the initial goal is to expend 2,500 kcal of energy per week**	**True**	35	15.8
	**False**	60	27.1
	**No idea**	126	57.0
**6: The MET (Metabolic equivalent) value is used to mathematically calculate the energy expended during exercise**	**True**	88	39.8
	**False**	5	2.3
	**No idea**	128	57.9
**7: In the initial phase of exercise, light muscular endurance exercises or moderate aerobic exercises can be prescribed**	**True**	151	68.3
	**False**	11	5.0
	**No idea**	59	26.7
**8: In healthy individuals, the duration of exercise should be gradually increased every 2–3 months**	**True**	120	54.3
	**False**	33	14.9
	**No idea**	68	30.8
**9: The intensity of exercise can be increased from moderate to vigorous in 20–30 minute sessions**	**True**	91	41.2
	**False**	29	13.1
	**No idea**	101	45.7
**10: Balance exercises should be recommended for patients aged 65 and older**	**True**	129	58.4
	**False**	14	6.3
	**No idea**	78	35.3
**11: It is appropriate for a patient with type 1 diabetes to exercise immediately after eating and right after insulin administration**	**True**	129	58.4
	**False**	22	10.0
	**No idea**	70	31.7
**12: Anaerobic exercise should be prescribed in diabetic patients with the presence of proliferative retinopathy and nephropathy**	**True**	72	32.6
	**False**	24	10.9
	**No idea**	125	56.6
**13: Aerobic exercise should be prescribed for patients with heart disease**	**True**	100	45.2
	**False**	35	15.8
	**No idea**	86	38.9
**14: Anaerobic activities help control high blood pressure**	**True**	61	27.6
	**False**	43	19.5
	**No idea**	117	52.9
**15: In patients with osteoarthritis, aerobic exercise should be prescribed first, followed by muscle-strengthening exercises**	**True**	17	7.7
	**False**	111	50.2
	**No idea**	93	42.1
**16**: **Exercise can be a trigger in asthma**	**True**	135	61.1
	**False**	32	14.5
	**No idea**	54	24.4

n: none.
